# Activity of erythrocyte antioxidant enzymes in healthy women depends on age, BMI, physical activity, and diet

**DOI:** 10.1186/s41043-022-00311-z

**Published:** 2022-08-17

**Authors:** Elżbieta Cecerska-Heryć, Klaudia Krauze, Angelika Szczęśniak, Aleksandra Goryniak-Mikołajczyk, Natalia Serwin, Daria Śleboda-Taront, Roksana Jacek, Rafał Heryć, Anna Michalczyk, Barbara Dołęgowska

**Affiliations:** 1 Department of Laboratory Medicine, Pomeranian Medical University of Szczecin, Powstanców Wielkopolskich 72, 70-111 Szczecin, Poland; 2 Department of Nephrology, Transplantology and Internal Medicine, Pomeranian Medical University of Szczecin, Powstancow Wielkopolskich 72, 70-111 Szczecin, Poland; 3 Department of Psychiatry, Pomeranian Medical University of Szczecin, Broniewskiego 26, 71-460 Szczecin, Poland

**Keywords:** Oxidative stress, Antioxidant enzymes, Obesity, Physical activity, Diet

## Abstract

**Introduction:**

Antioxidant enzymes protect the human body against the harmful effects of oxidative stress. The activity of antioxidant enzymes changes with age and depends on dietary nutrients such as fats and vitamins, which can have a significant impact on minimizing or exacerbating oxidative stress.

**Aim:**

To examine the effect of age, BMI, diet, physical activity, and smoking status on the activity of erythrocyte antioxidant enzymes catalase, glutathione reductase, glutathione peroxidase glutathione S-transferase, superoxide dismutase, and glutathione concentrations in healthy women.

**Material and methods:**

This study included 98 healthy women aged between 20 and 65 years. All women underwent anthropometric tests: body weight, height, hip, and waist circumference. Antioxidant activity in erythrocytes was measured by spectrophotometric methods.

**Results:**

Catalase activity increased significantly with age (*p* < 0.001), while superoxide dismutase activities and glutathione decreased with age (*p* = 0.008, *p* = 0.023, respectively). Women with a lower BMI (emaciation) had higher superoxide dismutase activity than those in the first degree of obesity (*p* = 0.009).

**Conclusions:**

(1) Increased catalase activity with age may signify a large amount of hydrogen peroxide resulting from malfunctioning antioxidant systems in old age. (2) A decline in superoxide dismutase activity with age may indicate inactivation of this enzyme, inappropriate SOD function in the presence of excessive amounts of hydrogen peroxide, and glycation of superoxide dismutase molecules. (3) A negative correlation between superoxide dismutase activity and the BMI index may indicate a decreased enzymatic activity in obese people.

**Supplementary Information:**

The online version contains supplementary material available at 10.1186/s41043-022-00311-z.

## Introduction

The oxygen that we breathe is reduced in the body, resulting in a water molecule. The products of incomplete reduction of the oxygen molecule are called reactive oxygen species (ROS). These molecules or free radicals quickly form chain reactions, reacting with proteins, sugars, lipids, and nucleic acids in cells, leading to the formation of free radical products [1].

Physiologically, ROS are formed in the respiratory chain, during purine nucleotide metabolism, and in the microsomal hydroxylation cycle, in a reaction taking place with oxidoreductases. Their task is to induce cell differentiation and apoptosis, influence the synthesis, release, or inactivation of the endothelial vasodilator endothelial-derived relaxing factor (EDRF), extend or contract the wall of blood vessels, and stimulate glucose transport into cells, or serotonin into platelets. ROS also take part in immunological processes [1]. The most important enzymes involved in the neutralization of ROS are superoxide dismutase (SOD), catalase (CAT), glutathione peroxidase (GPx), glutathione reductase (GR), glutathione S-transferase (GST), and glucose-6-phosphate dehydrogenase (G6PDH). Among the non-enzymatic antioxidants, we studied glutathione (GSH), which directly and indirectly binds the majority of enzymatic antioxidants [2, 3].

The amount of ROS synthesis correlates with the activity of intracellular antioxidant systems [3]. The resulting generation of ROS can lead to undesirable effects on the function of the body, including metabolic disorders and changes in nucleic acids. Oxidative stress occurs when there is a lack of balance between the production and removal of ROS, and the antioxidant system aims to rebalance ROS levels.

The activity of antioxidant enzymes may change with age. In recent years, processes connected with the aging of the body with the action of reactive oxygen species have been increasingly investigated. In order to prevent the accumulation of ROS, the body has developed antioxidant mechanisms. Changes in their activity and concentration depend on the race, sex, organ, and location of the subcellular enzyme. The reduction in their activity observed with age is caused by the direct or indirect modification of enzyme molecules by ROS. In turn, increasing their activity should be treated as a compensation response to overproduction of free oxygen radicals. With age, GSH synthesis decreases as a result of the reduced availability of cysteine and methionine and reduced activity of γ-glutamyl cysteine synthetase and cystathionase on the one hand, as well as increased GSH consumption in reactions with free radicals, which are generated in excessive amounts [4].

In the literature, you can also find reports on the nutrients contained in the diet (fats, vitamins) that can have a significant impact on the minimization or intensity of oxidative stress. For years, there has also been a discussion on whether physical activity or smoking affects the activity of antioxidant systems in the body and, if so, how these factors affect antioxidant activities. Current research also indicates that the antioxidant systems undergo significant changes in response to acute and chronic exercise. This is also related to age, likely because physical activity usually decreases in older people. Acute exercise may increase the activity of some antioxidative enzymes in various tissues, but the mechanism of this activation is unclear. Exercise training has little effect on liver enzymes or cardiac muscle systems, but it can cause adaptive reactions in antioxidant enzymes within skeletal muscle, particularly in GPx. These findings suggest that aging, physical exercise, and diet may impose oxidative stress on the body [2]. The purpose of this work is to answer the above questions.

## Materials and methods

### Ethical approval and consent

The Bioethical Commission at the Pomeranian Medical University in Szczecin approved the research carried out (no KB-0012/36/11). All participants were informed about the purpose and scope of the study and gave their consent to donate samples and for the resulting data to be published.

### Study group

The study covered 98 healthy women between the ages of 20 and 65. Their health status was confirmed on the basic morphological and biochemical tests (total cholesterol, triglycerides, low-density lipoprotein (LDL), high-density lipoprotein (HDL), total protein, albumin, glucose, uric acid) in the Department of Laboratory Diagnostics at the Independent Public Clinical Hospital No. 2 in Szczecin. No results were found that depart from the standards adopted at the Central Laboratory and in methodologies from producers (Biomaxima, Lublin, Poland). All women underwent anthropometric tests: body weight, height, hip, and waist circumference. A survey was also carried out to assess the diet and physical activity of the women surveyed and whether they smoked cigarettes. A questionnaire regarding the occurrence of chronic diseases was also asked in the survey, and all subjects studied denied the presence of chronic disease. Healthy volunteers did not have to be on a special diet or show increased physical strength before and during the study. Detailed data on age, body mass index (BMI), waist-to-hip ratio (WHR), physical activity, diet, and smoking are presented in Tables 1, 2, 3 and 4. The BMI index criteria were: 16–16.99—emaciation, 17–18.99—underweight, 19–24.99—standard, 25–29.99—overweight, 30–34.99—1st degree of obesity. All women agreed to participate in the study. The Bioethical Commission approved the study at the Pomeranian Medical University in Szczecin.

**Table 1:** Characteristics of the study group in terms of age, BMI and WHR (Avg—arithmetic mean, SD—standard deviation, Min—minimum value, Max—maximum value) and physical activity

	*n*	%
*Age range*		
Age		
20–35 years	61	62.2
36–45 years	17	17.3
46–55 years	11	11.2
56–65 years	9	9.2
*Indicator*		
BMI		
Emaciation	3	3.1
Underweight	11	11.2
Normal	67	68.4
Overweight	13	13.3
I degrees of obesity	4	4.1
*Physical activity*		
No	56	57.1
Yes	42	42.9
*Number of hours per week*		
0	56	57.1
1	10	10.2
2	15	15.3
3	10	10.2
4	2	2.0
5	5	5.1

Indicator	Avg	SD	Min	Max
BMI	22.3	3.5	16.0	32.6
WHR	0.8	0.1	0.7	0.9

*n* number of people, *BMI* body mass index, *WHR* waist-hip ratio

**Table 2:** Characteristics of the study participants in basic biochemical parameters and blood morphology (mean ± standard deviation, median—lower and upper quartile)

Parameter	Group
Concentration of creatinine [mg/dl]	0.2 ± 0.16 0.1 (0.05; 0.18)
Concentration of triglycerides [mg/dl]	151.29 ± 5.17 168.69 (164.95; 170.09)
Concentration of albumin [g/dl]	4.21 ± 0.31 4.27 (4.15; 4.51)
Concentration of total protein [g/dl]	2.19 ± 0.19 2.24 (2.12; 2.38)
Concentration of cholesterol [mg/dl]	146.2 ± 28.75 130.43 (117.39; 160)
Concentration of glucose [mg/dl]	90.47 ± 21.55 92.73 (82.8; 108.6)
Concentration of uric acid [mg/dl]	6.98 ± 3.41 7.14 (5.82; 9.29)
Red blood cells [M/µl]	4.7 ± 0.5 4.4 (4.2; 5.5)
Hemoglobin [mmol/l]	8.6 ± 1.5 7.1 (7.0; 9.0)
Hematocrit [%]	37 ± 1.8 37.2 (36.5; 38.0)
Mean corpuscular volume (MCV) [fl]	87 ± 3.0 85.0 (84.0; 89.0)
Mean corpuscular hemoglobin (MCH) [pg]	29 ± 1.2 28.0 (27.1.0; 29.0)
Mean corpuscular hemoglobin concentration (MCHC) [g/dl]	34 ± 1.0 33.0 (32.0;35.0)

**Table 3:** Antioxidant enzymes activity and GSH concentration in particular age groups (Av—arithmetic mean, SD—standard deviation, Min—minimum value, Max—maxim um value)

Parameter	20–35 years		36–45 years		46–55 years		56–65 years		*p*
	Avg	SD	Avg	SD	Avg	SD	Avg	SD	
SOD [U/mgHb]	0.37	0.20	0.35	0.25	0.29	0.17	0.20	0.09	0.062
CAT [U/mgHb]	0.24	0.13	0.39	0.21	0.46	0.16	0.36	0.16	** < 0.001**
GPx [U/gHb]	0.05	0.04	0.04	0.02	0.03	0.01	0.03	0.02	0.684
GSH [µmol/gHb]	9.93	1.87	10.90	3.47	9.17	2.00	8.40	1.72	0.107
GST [U/gHb]	0.04	0.03	0.03	0.02	0.06	0.06	0.03	0.02	0.230
GR [U/gHb]	8.67	14.51	5.23	4.09	3.47	2.86	7.35	7.84	0.524

The results in bold are statistically significant

*p*—statistical significance of the relationship between age of healthy volunteers and activity of antioxidant enzymes and concentration of GSH—Kruskal–Wallis ANOVA

**Table 4:** Antioxidant enzymes activity and GSH concentration in particular BMI groups (Avg—arithmetic mean, SD—standard deviation, Min—minimal value, Max—maximum value)

Parameter	Emaciation		Underweight		Normal		Overweight		1st degree of obesity		*p*
	Avg	SD	Avg	SD	Avg	SD	Avg	SD	Avg	SD	
SOD [U/mgHb]	0.48	0.15	0.52	0.23	0.31	0.18	0.37	0.23	0.23	0.14	**0.009**
CAT [U/mgHb]	0.33	0.02	0.25	0.09	0.31	0.19	0.30	0.14	0.35	0.24	0.806
GPx [U/gHb]	0.09	0.06	0.06	0.04	0.04	0.03	0.05	0.04	0.05	0.05	0.540
GSH [µmol/gHb]	12.12	2.23	10.37	1.39	9.67	2.47	9.62	1.56	11.13	2.72	0.103
GST [U/gHb]	0.05	0.02	0.05	0.03	0.04	0.03	0.04	0.06	0.04	0.02	0.438
GR [U/gHb]	5.09	4.09	4.93	4.57	8.20	13.94	7.00	6.67	3.06	2.40	0.632

The results in bold are statistically significant

*p*—statistical significance of the relationship between BMI, antioxidant enzyme activity and GSH concentration

### Samples

Venous blood was collected in 5 ml tubes and allowed to clot before centrifugation (10 min, 3000 rpm, 20 °C), and the serum was transferred to subsequent tubes and frozen at − 80 °C until the analyses were performed. Additional venous blood samples were collected into 5 ml tubes containing an anticoagulant (K3EDTA), and morphological parameters of the blood were assessed. The blood was centrifuged (10 min, 3000 rpm, 20 °C), and the plasma was transferred to another tube and frozen at − 80 °C until analyses were performed. The remaining red blood cells were rinsed three times with 0.9% NaCl. After the last rinsing and removal of NaCl, the erythrocytes were transferred to appropriately labeled tubes and frozen at − 80 °C until analyses were performed [3, 5]. The analysis of the activity of antioxidant enzymes was carried out on an ongoing basis. The samples were not stored for more than 3 months at − 80 °C.

### Activity of antioxidant enzymes

The activity of the antioxidant enzymes sodium dismutase (SOD), catalase (CAT), glutathione peroxidase (GPx), glutathione reductase (GR), glutathione (GSH) was measured by spectrophotometric methods.

#### Determination of superoxide dismutase activity in erythrocytes

Reagents: adrenaline, carbonate buffer, chloroform, ethanol. Reagents were purchased from Sigma-Aldrich (Poznań, Poland).

An extract was prepared by centrifuging 300 μl water, 200 μl chloroform/ethanol, and 250 μl hemolysate II. The tested and blank samples were determined. 1425 μl carbonate buffer, 25 μl of obtained extract, and 50 μl of adrenaline were added to the test sample. On the other hand, 1475 µl of carbonate buffer and 25 µl of obtained extract were added to the blank. Then, both samples were incubated in a water bath for 3 min and the extinction measured at 320 nm for 3 min [3, 5, 6].

#### Determination of catalase activity in erythrocytes

Reagents: phosphate buffer and hydrogen peroxide. Reagents were purchased from Sigma-Aldrich (Poznań, Poland).

Hemolysate IV was made by adding 10 ml of hemolysate II buffer to 5000 ml. At the same time, both the tested and blank samples were determined. 1000 µl of hemolysate IV and 500 µl of hydrogen peroxide were added to the test sample, while an additional 500 µl of phosphate buffer was added to the blank. Extinction was measured at 240 nm over 30 s [3, 5, 6].

#### Determination of glutathione peroxidase activity in erythrocytes

Reagents: phosphate buffer, glutathione reductase in phosphate buffer, reduced glutathione, NADPH+H+, tert-butyl hydroxide (T-BOOH). Reagents were purchased from Sigma-Aldrich (Poznań, Poland).

250 μl transforming reagent was added to 500 μl hemolysate III and incubated for 5 min at room temperature. Then, 250 μl of hemolysate with transforming reagent, 50 μl of reduced glutathione, 50 μl of NADPH+H+, and 50 μl of glutathione reductase were added to 550 μl of phosphate buffer, followed by incubation for 10 min in a water bath. After incubation, 50 µl of T-BOOH was added and the extinction decline at 340 nm was measured against distilled water as a blank [3, 5, 6].

#### Determination of glutathione concentration in erythrocytes

Reagents: precipitation solution (glacial metaphosphoric acid, disodium/dipotassium EDTA, bidistilled water), DTNB, phosphate buffer pH 7.9. Reagents were purchased from Sigma-Aldrich (Poznań, Poland).

50 μl hemolysate II was added to 450 μl of distilled water. 750 ml of the precipitation solution was mixed with the hemolysate and incubated for 5 min at 4 °C, followed by centrifugation (550*g*, 10 min). 250 ml of supernatant was added to 1 ml of phosphate buffer followed by 125 ml of DTNB solution and incubated 15 min at 4 °C. Extinction was determined at *λ* 412 nm at 25 °C [3, 5, 6].

#### Determination of glutathione transferase activity in erythrocytes

Reagents: phosphate buffer, GSH solution, CDNB solution (1-chloro-2,4-dinitrobenzene). Reagents were purchased from Sigma-Aldrich (Poznań, Poland).

Hemolysate II with a hemoglobin concentration of 5 g/dl diluted tenfold with distilled water (9:1) was prepared. Each sample was then assayed separately by adding 850 μl phosphate buffer, 50 μl GSH solution, and 50 μl CDNB solution. The extinction increase at 340 nm was measured against distilled water as a blank [3, 5, 6].

#### Determination of glutathione reductase activity in erythrocytes

Reagents: diluted erythrocyte hemolysate (1250 µl water and 62.5 µl erythrocytes), triethanolamine buffer (EDTA, pH 7.5), diluted RI working reagent (900 µl EDTA and 100 µl RI), working reagent RII. Reagents were purchased from Sigma-Aldrich (Poznań, Poland).

25 ml of hemolysate was added to 1 ml of RI solution and incubated 5 min at 30 °C. Then, 0.1 ml RII reagent was added and extinction measured at *λ* 340 nm over 5 min at 30 °C [3, 5, 6].

### Statistical analysis

Descriptive statistics (arithmetic mean, standard deviation (SD), minimum and maximum values) of body mass index (BMI), waist-to-hip ratio (WHR), antioxidant enzymes, and GSH are reported. The Shapiro–Wilk test was used to check the assumption of normality for the data on GSH concentration and enzyme activity, which in the case of most variables showed a non-normal distribution of parameters. Differences in enzyme activity and GSH levels in the age groups and BMI categories were analyzed. The nonparametric Kruskal–Wallis ANOVA test was used for intergroup comparisons. The analysis also included differences in enzyme activity and GSH concentration depending on physical activity and cigarette smoking. The nonparametric Mann–Whitney U test was used for intergroup comparisons. The correlation strength between the parameters was measured using Spearman's rank correlation.

In order to determine a multifactorial evaluation of relationships between the parameters studied, a linear multiple regression model was used. The antioxidative enzymes and GSH concentrations were analyzed as dependent variables. The age and BMI of healthy volunteers were introduced as independent variables.

Statistical analysis of the results was carried out using Statistica PL 13 statistical program (StatSoft). *p* values ≤ 0.05 were assumed to be statistically significant.

## Results

**Fig. 1:**
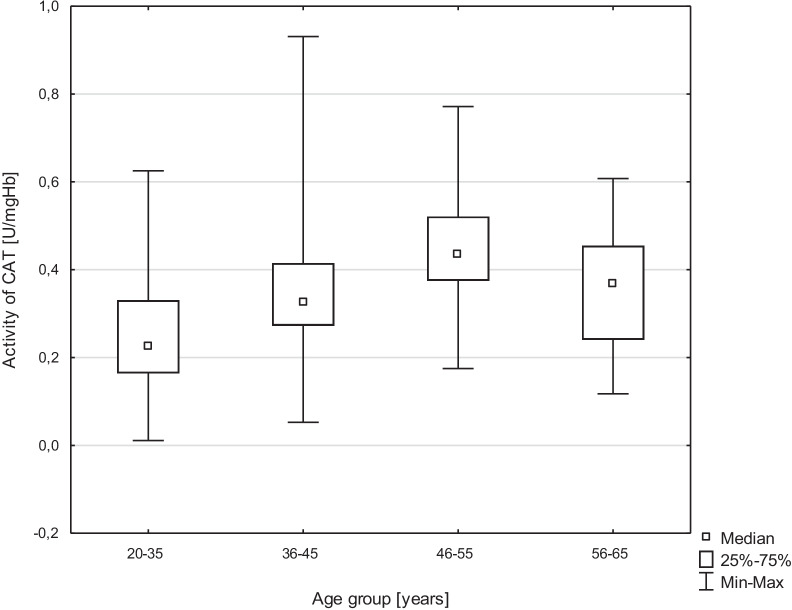
Kruskal–Wallis ANOVA analysis of age influence on CAT activity (*p* < 0.001) CAT—catalase

This study analyzed the relationship between the age of healthy volunteers and the activity of antioxidative enzymes and the concentration of GSH. There was a significant relationship between age and CAT activity (*p* < 0.001), with the highest CAT activity in the 46–55 age group, and the lowest in the youngest group (Fig. 1). The results are shown in Table 3.

**Fig. 2:**
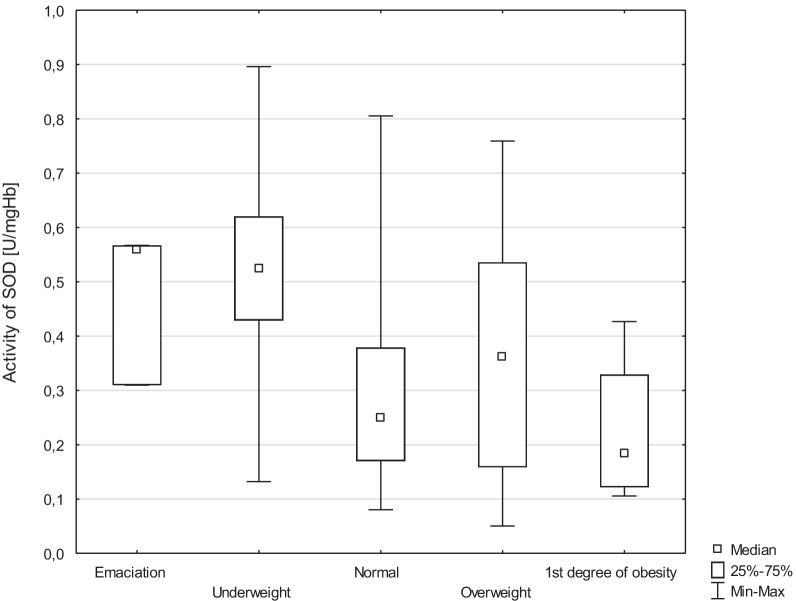
Kruskal–Wallis ANOVA analysis of age influence on SOD activity (*p* = 0.009) SOD—sodium dismutase

This study also analyzed the relationship between individual age groups of healthy volunteers and the activity of antioxidative enzymes and GSH concentrations. A significant relationship was found between CAT activity and the age range of 20–35 years and 36–45 years (*p* = 0.002). Statistical significance was also demonstrated in the case of GST activity (*p* = 0.047) in the same age ranges. Comparison of the age ranges of 20–35 years and 46–55 years showed a significant result in the activity of CAT (*p* < 0.001) (Fig. 2; Tables 5, 6, 7).

**Table 5:** Activity of antioxidant enzymes and concentration of GSH in age groups 20-35 and 36-45 years

	Median 20–35 years	Median 36–45 years	p
SOD [U/mgHb]	0.34	0.22	0.438
CAT [U/mgHb]	0.23	0.33	0.002
GPx [U/gHb]	0.04	0.04	0.743
GSH [µmol/gHb]	9.84	9.89	0.549
GST [U/gHb]	0.04	0.03	0.047
GR [U/gHb]	3.51	4.00	0.990

*p* - statistical significance of the relationship between individual age groups, antioxidant enzyme activity and GSH concentration - Kruskal-Wallis ANOVA

**Table 6:** Activity of antioxidant enzymes and concentration of GSH in age groups 20-35 and 46-55 years

	Median 20–35 years	Median 46–55 years	p
SOD [U/mgHb]	0.34	0.22	0.193
CAT [U/mgHb]	0.23	0.33	< 0.001
GPx [U/gHb]	0.04	0.04	0.402
GSH [µmol/gHb]	9.84	8.83	0.406
GST [U/gHb]	0.04	0.03	0.863
GR [U/gHb]	3.51	2.70	0.259

*p* - statistical significance of the relationship between individual age groups, antioxidant enzyme activity and GSH concentration - Kruskal-Wallis ANOVA

**Table 7:** Activity of antioxidant enzymes and concentration of GSH in age groups 20-35 and 56-65 years

	Median 20–35 years	Median 56–65 years	p
SOD [U/mgHb]	0.34	0.18	0.008
CAT [U/mgHb]	0.23	0.37	0.027
GPx [U/gHb]	0.04	0.04	0.493
GSH [µmol/gHb]	9.84	8.37	0.023
GST [U/gHb]	0.04	0.03	0.250
GR [U/gHb]	3.51	3.50	0.466

*p* - statistical significance of the relationship between individual age groups, antioxidant enzyme activity and GSH concentration - Kruskal-Wallis ANOVA

Statistical significance was also demonstrated by comparing the age groups of 20–35 years and 56–65 years for SOD activity (*p* = 0.008), CAT (*p* = 0.027), and GSH concentration (*p* = 0.023).

The differences in enzyme activity in individual BMI categories were analyzed. A significant relationship was found between SOD activity and BMI (*p* = 0.009). The highest activity of SOD was observed in subjects who were classified by BMI as underweight, and the lowest activity was observed in people with 1st degree of obesity (Table 4).

The study also analyzed the relationship between individual BMIs of healthy volunteers and the activity of antioxidant enzymes and GSH concentration. A significant relationship was demonstrated in the case of SOD activity in people who were underweight and with normal body mass (*p* = 0.006). Statistical significance was also demonstrated in the case of this enzyme by comparing people with the 1st degree of obesity and those who were underweight (*p* = 0.036). Detailed results are presented in Table 8.

**Table 8:** Antioxidant enzymes activity and GSH concentration in people with underweight normal body mass, 1st degree of obesity

	Median underweight	Median normal	*p*
SOD [U/mgHb]	0.53	0.26	**0.006**
CAT [U/mgHb]	0.26	0.28	0.417
GPx [U/gHb]	0.07	0.04	0.201
GSH [µmol/gHb]	10.18	9.35	0.077
GST [U/gHb]	0.04	0.04	0.332
GR [U/gHb]	3.00	3.24	0.397

	Median 1st degree of obesity	Median underweight	*p*
SOD [U/mgHb]	0.19	0.53	**0.036**
CAT [U/mgHb]	0.35	0.26	0.556
GPx [U/gHb]	0.04	0.07	0.794
GSH [µmol/gHb]	10.92	10.18	0.647
GST [U/gHb]	0.05	0.04	0.844
GR [U/gHb]	1.99	3.00	0.948

The results in bold are statistically significant

*p*—statistical significance of the relationship between underweight and normal BMI index and people with 1st degree of obesity and underweight, and the activity of antioxidant enzymes and concentration of GSH—Kruskal–Wallis ANOVA

There were no significant differences in enzyme activity and GSH concentration depending on physical activity levels and cigarette smoking (Additional file 1: Tables S3 and S4).

The correlation between GSH concentration and enzyme activity, age, waist-to-hip ratio, body mass index, number of hours of physical activity, and cigarette smoking was analyzed. There was a significant negative correlation between age and SOD activity (*p* = 0.007) and GST (*p* = 0.025). The activity of these enzymes decreases with age. In contrast, a significant positive correlation was demonstrated between age and CAT activity. The activity of CAT increases with age. Detailed results are presented in Table 9.

**Table 9:** Correlation coefficients between activity antioxidant enzymes and GSH concentration and age and BMI

Antioxidant	Age	*p*
SOD [U/mgHb]	** − 0.27**	**0.007**
CAT [U/mgHb]	**0.39**	**0.000**
GPx [U/gHb]	− 0.06	0.570
GSH [µmol/gHb]	− 0.13	0.212
GST [U/gHb]	** − 0.23**	**0.025**
GR [U/gHb]	− 0.02	0.881

Antioxidant	BMI	*p*
SOD [U/mgHb]	** − 0.25**	**0.014**
CAT [U/mgHb]	0.05	0.642
GPx [U/gHb]	− 0.12	0.241
GSH [µmol/gHb]	− 0.11	0.300
GST [U/gHb]	− 0.12	0.246
GR [U/gHb]	0.16	0.120

The results in bold are statistically significant

*p*—statistical significance of the relationship between age and BMI and activity of antioxidant enzymes and GSH concentration—Spearman's rank correlation analysis

There was no correlation between WHR and the activity of antioxidant enzymes and GSH concentration (Table 6). There was a significant negative correlation between BMI and SOD activity (*p* = 0.014) (Table 9). No correlations were obtained between antioxidant enzyme activities or GSH concentrations and physical activity levels (taking into account the number of hours of effort per week) and the activity of antioxidant enzymes and GSH concentration. There was no significant correlation between the activity of antioxidant enzymes and GSH concentration and smoking.

Multivariate regression analysis was performed. The influence of age, WHR, and BMI (independent variables) on the activity of individual enzymes (dependent variable) was examined.

In the case of SOD, it has been shown that age affects its activity in an average of 5%. With increasing age, the activity of SOD decreases by an average of 0.0033 U/mgHb per year. In the case of CAT, it was shown that age affects its activity in an average of 17%. With increasing age, CAT activity increases by an average of 0.0057 U/mgHb during the year (Table 10).

**Table 10:** Results of multivariate regression analysis in relation to SOD and CAT

Dependent variable	Independent variable	*β*0	*β*1	*p*	*R*^2^	*F*
SOD	Age	** − 0.217**	** − 0.003**	**0.041**	**0.050**	2.721
	WHR	− 0.021	− 0.069	0.829		
	BMI	− 0.120	− 0.006	0.254		
CAT	Age	**0.437**	**0.005**	**0.000**	0.170	7.655
	WHR	0.163	0.452	0.080		
	BMI	− 0.093	− 0.004	0.343		

The results in bold are statistically significant

*β*—standardized coefficient in the regression equation; *β*1—unknown (and determined) regression coefficient; *β*0—word free; *R*^2^—coefficient of determination; *p *value of materiality coefficient; SOD—superoxide dismutase; and CAT—catalase

There were no significant correlations between serum biochemical parameters and the activity of antioxidant enzymes in the study group.

A significant correlation was found between the activity of CAT and diet, specifically the reduction of fish (*r* =  − 0.202; *p* value = 0.046) and dairy (*r* =  − 0.209; *p* value = 0.038) consumption. Women who tried not to eat fish and dairy had lower catalase activity (Table 11).

**Table 11:** Correlation coefficients between activity of CAT activity diet

Antioxidant	CAT [U/mgHb]	*p*
Limiting the consumption of fish	** − 0.202**	**0.046**
Limiting dairy consumption	− 0.209	0.039

The results in bold are statistically significant

*p*—statistical significance of the relationship between diet and activity of CAT—Spearman's rank correlation analysis

Multivariate regression analysis was performed. The influence of physical activity, smoking cigarettes, and diet (independent variables) on the activity of individual enzymes (dependent variable) was examined. The model has not been shown to influence the activity of the tested enzymes. Only single variables had a significant effect, and so in the case of catalase, it was cigarette smoking and dairy reduction, which was partially confirmed by the results of the correlation analysis; in the case of GPx, these were physical activity and restriction of fish consumption; and in the case of GSH, a reduction in the consumption of bread (Table 12).

**Table 12:** Results of multivariate regression analysis in relation to CAT, GPx, and GSH

Dependent variable	Independent variable	*β*0	*β*1	*p*	*R*^2^	*F*
CAT	Physical activity	− 0.217	0.117	0.07	0.07	1.851
	Smoking	** − 0.252**	**0.104**	**0.017**		
	Limiting the consumption of sugar	− 0.027	0.119	NS		
	Limiting the consumption of bread	0.198	0.124	NS		
	Limiting the consumption of fish	− 0.065	0.112	NS		
	Limiting the consumption of meat	− 0.06	0.125	NS		
	Limiting the consumption of vegetables	0.081	0.1	NS		
	Limiting the consumption of fruit	0.256	0.126	NS		
	Limiting the consumption of diary	** − 0.241**	**0.102**	**0.02**		
	Limiting the consumption of fats	0.109	0.127	NS		
GPx	Physical activity	**0.302**	**0.119**	**0.01**	0.165	1.48
	Smoking	− 0.061	0.106	NS		
	Limiting the consumption of sugar	0.108	0.121	NS		
	Limiting the consumption of bread	− 0.03	0.127	NS		
	Limiting the consumption of fish	** − 0.282**	**0.114**	**0.015**		
	Limiting the consumption of meat	0.105	0.127	NS		
	Limiting the consumption of vegetables	− 0.038	0.102	NS		
	Limiting the consumption of fruit	− 0.287	0.156	NS		
	Limiting the consumption of diary	− 0.068	0.104	NS		
	Limiting the consumption of fats	− 0.228	0.129	NS		
GSH	Physical activity	0.033	0.120	NS	0.343	1.14
	Smoking	− 0.046	0.107	NS		
	Limiting the consumption of sugar	− 0.079	0.122	NS		
	Limiting the consumption of bread	**0.27**	**0.128**	**0.038**		
	Limiting the consumption of fish	− 0.1	0.116	NS		
	Limiting the consumption of meat	− 0.056	0.129	NS		
	Limiting the consumption of vegetables	− 0.005	0.104	NS		
	Limiting the consumption of fruit	− 0.01	0.234	NS		
	Limiting the consumption of diary	0.12	0.106	NS		
	Limiting the consumption of fats	0.049	0.131	NS		

The results in bold are statistically significant

*β*—standardized coefficient in the regression equation; *β*1—unknown (and determined) regression coefficient; *β*0—word free; *R*^2^—coefficient of determination; *p *value of materiality coefficient; CAT—catalase; GPX—glutathione peroxidase; and GSH—glutathione

## Discussion

Changes in the activity of antioxidant enzymes and GSH level in relation to age are commonly described in many articles and scientific publications. However, the specificity of the change in activity of these enzymes and GSH in relation to physical activity, diet, or smoking is less known.

The literature describes the importance of oxidative stress and the reduced efficiency of repair processes in the process of aging. The most visible effects of these pathological conditions are revealed in DNA. DNA damage caused by the action of reactive oxygen species can lead to the formation of mutations, which in turn may be a cause of cancer development. Therefore, with increasing age, humans experience a higher incidence of various diseases, mainly including cancers but also neurodegenerative diseases and atherosclerosis, among other disease. Well-functioning repair systems remove damage and prevent harmful changes in cells. Unfortunately, with age, they are weakened, which contributes to an increase in the number of damaged cells [7].

Changes related to the aging of the body and increasingly becoming associated with the operation of ROS. In order to avoid the accumulation of ROS, the body has developed mechanisms of antioxidant defense, which include, among others, the action of enzymes such as CAT, SOD, GST, GPx, or non-enzymatic antioxidants such as GSH. Changes in their activity and concentration depend on the organ or subcellular location of the enzyme, as well as race and sex, and other variables. The reduction of their activity and concentration with age is caused by the modification of the enzyme molecule, which is caused directly or indirectly by ROS. Increasing their activity, on the other hand, should be treated as a compensatory response to overproduction of reactive oxygen species. With age, there may also be a decrease in GSH synthesis due to the much lower availability of methionine and cysteine, and the activity of γ-glutamyl cysteine synthetase and cystathionase, as well as increased GSH consumption in reactions with ROS, produced in too large quantities [4].

An important enzyme involved in the defense of the body against oxidative stress is CAT. CAT reacts with hydrogen peroxide (H_2_O_2_) to form water and molecular oxygen, as well as compounds such as methanol, ethanol, formic acid, and phenol. CAT thus protects the body against the effects of hydrogen peroxide produced in cells and is one of the most efficient enzymes in the fight against oxidative stress [8]. This study analyzed differences in the levels of antioxidant enzymes in particular age groups. A significant increase in CAT activity with age was demonstrated. The highest activity was observed in the 46–55 age group. These results also seem to be confirmed by multivariate regression analysis, which showed an increase in CAT activity with increasing age in healthy volunteers (an average increase of 0.0057 U/mgHb per year). These data support the generally accepted hypothesis regarding the increase in the activity of CAT in an aging organism [9]. The increase in the activity of this enzyme may also be related to the activity of GST. There was a significant (*p* = 0.047) decline in its activity among the elderly. These enzymes, due to their function to reduce peroxides, can complement one another. Therefore, when one of these enzymes increases, the growth of the other is inhibited [5]. Nandi et al. analyzed the role of catalase in age-related diseases such as Alzheimer's, Parkinson's, and diabetes. They found that CAT plays a role in the pathophysiology of these diseases. In the future, it could be used as a therapeutic agent, which is of great importance for increasingly aging societies. However, it requires a lot of clinical research [10].

Another important enzyme in antioxidant defense is GPx. This enzyme reduces both inorganic peroxides, e.g., H_2_O_2_, as well as organic peroxides (ROOH) to form selenic acid as an intermediate [11, 12]. Peroxidase has a greater affinity for hydrogen peroxide than CAT; therefore, it performs a more important function in most physiological situations, when the amount of hydrogen peroxide formed is not too high. Insufficient CAT activity is therefore compensated by an increase in GPx activity, and conversely, reduced peroxidase activity is compensated by an increase in CAT activity [3, 6]. In the current studies reported here, no increased GPx activity was observed, in any age range, as well as in dependence from BMI. This may be related to the greater activity of CAT, which, despite its lower affinity for H_2_O_2_, performs this reaction with greater efficiency. This is evidenced by the increase in the activity of CAT with age. However, this does not confirm that peroxidase is more active in physiological situations. Very interesting studies by Lapenn et al. found that aging impairs the enzymatic reactive aldehyde-detoxifying capacity and GPx activity of the human arterial tissue, eventually favoring vascular oxidative stress interesting. This could explain the lower activity of GPx than CAT in older people [13].

Olędzki et al. compared the activity of SOD and CAT in erythrocytes taken from young healthy people (aged 20–29) and older individuals (> 60 years of age). They reported reduced SOD activity in the older patients. The study also showed significant differences in the activity of SOD between the group of the youngest and the oldest women. According to the study reported here, the SOD activity decreases by an average of 0.0033 U/mgHb per year. In the case of CAT, decreased activity in the elderly was observed in relation to younger people, which is in opposition to the data presented here. Based on these results, it was found that the antioxidant defense level of erythrocytes, during their more than 100-day duration, is not stable in both young and old people and that it decreases in the physiological aging process [14].

The reduction of SOD activity and the increase in CAT activity among aging women can be explained by enzyme inactivation by excess hydrogen peroxide, as well as by glycation of SOD molecules or reactions with lipid peroxidation products, the intensity of which increases with age [6]. However, we can only assume this on the basis of the literature data and the results of CAT and SOD activity obtained by us. These results, however, require confirmation in further studies. A significant increase in the activity of SOD was found in people who were underweight and a significant decrease in people with the first degree of obesity. This enzyme is an antioxidant that catalyzes the superoxide anion radical dismutation reaction for hydrogen peroxide and molecular oxygen, thus contributing to effective defense against oxidative stress [9, 15].

Karolkiewicz and colleagues evaluated the relationship between body mass and insulin resistance parameters, as well as the relationship between body weight and oxidative stress markers in older women. The population studied consisted of 34 women aged 60–90 who were divided into three subgroups based on their BMI: normal weight, overweight, and obese. The total antioxidative status (TAS), the concentration of substances reacting with thiobarbituric acid, and the level of protein were measured in the plasma C-reactive protein (CRP). However, the concentration of GSH and GPx activity was determined in hemolysate of red blood cells. The results did not reveal any significant differences between the three groups of women surveyed in relation to antioxidant status parameters. There was also no disturbed balance between oxidants and antioxidants [16]. However, the relationship in the case of SOD has not been studied; therefore, it is unknown what the reaction would have been with this enzyme. The current study reported here also did not show statistical significance with respect to GSH concentration and GPx activity.

The impact of diet and increased plant sterol supply on the parameters of oxidative stress in the group of obese women was examined previously by Stelmach-Mardas and co-workers. The study covered 101 women with a BMI > 30 kg/m^2^. They were divided into two groups: the study group (60 women) and the control group (41 women). Anthropometric measurements were made, such as body weight, height, waist, and hip circumference. Parameters were calculated, including the value of the body mass index and waist–hip index and percentage of adipose tissue. The lipid profile and oxidative stress parameters (malondialdehyde (MDA), oxidized protein, hydroxydioxides, CAT, SOD) were measured by enzymatic-colorimetric methods. The results showed significant (*p* < 0.05) differences between the examined groups in relation to oxidative stress parameters after supplementing the diet with plant sterols [17].

The results showed significant (*p* < 0.05) differences between the studied groups in the parameters of oxidative stress after supplementing the diet with plant sterols [17]. In the study group showed a decrease in the amount of hydroxydioxides (*p* = 0.0011) and a tendency to reduce the activity of malondialdehyde (*p* = 0.0018), oxidized protein, and above all SOD (*p* = 0.0004). Xiao-Liao et al. conducted a study for SOD in a group of 136 young- and middle-aged men. The men were divided into three groups based on BMI: group I—obese (43 people), group II—overweight (46 people), and a control group with normal weight (47 people). Statistical analysis was performed, the results of which, in relation to oxidative stress parameters, showed a significant decrease in the activity of MDA and SOD in overweight and obese people [18]. With regard to our current study, it can be assumed that anthropometric features are also strongly related to the activity of individual antioxidant enzymes. It is true that healthy volunteers, before and during the study, did not have to be on a special diet, as was the case in the study by Stelmach-Mardas et al., although SOD activity was similar. The decrease in the activity of SOD in the case of people with 1st degree of obesity and its increase in underweight individuals have also been demonstrated.

Mohensi et al., on the other hand, conducted a study on obese children and children with average body weight aged 8–16 years. They demonstrated that Mn-SOD and CAT gene expression was significantly lower in the obese group compared with the control group (*p* < 0.01). Also, a positive correlation was observed between the gene expression of Mn-SOD and CAT and BMI, fasting blood sugar, insulin resistance, low-density lipoprotein-cholesterol (LDL-C), triglycerides, and systolic blood pressure. Induction of antioxidants, especially Mn-SOD and CAT, can reduce oxidative stress and prevent the complications of obesity in children [19].

Obesity is defined as the body mass exceeding the upper limit of physiological needs caused by excessive fat accumulation [20, 21]. The human body has brown and white adipose tissue. White adipose tissue contains fibroblasts, adipocyte, and macrophages, which are characterized by an obvious heterogeneity associated with their location, e.g., subcutaneous or visceral. In addition, white adipose tissue is not only the tissue that stores energy in the body, but also has an endocrine, paracrine, and autocrine function [20, 22]. Bioactive substances secreted by adipose tissues are called adipocytes or adipocytokines (including leptin and adiponectin) [20, 23]. The latter two can increase energy consumption, insulin sensitivity, and fatty acid oxidation, while leptin can suppress appetite and fat aggregation. In addition, adipokines increase the production of reactive oxygen species and cause oxidative stress, respectively. Therefore, obesity correlates significantly with the growth of oxidative stress markers [20, 24].

Of course, diet should be taken as a factor having a significant impact on the increase or decrease in the activity of individual antioxidant enzymes and GSH concentration. A diet rich in fat reduces the activity and concentration of antioxidants. On the contrary, eating a large amount of fruits and vegetables increases the activity and concentration of antioxidants [12]. In this case, attention should be paid to the characteristics of the women surveyed in Table 3. Almost 70% of women limited fat intake, and the majority did not limit consumption of fruit and vegetables in their diet. This applies mainly to older people, because the diet changes with age and health status, which often determines the consumption of proper foods in the elderly.

Epidemiological studies indicate that fruit and vegetables have a protective effect against diseases typical of old age, such as joint degeneration, cardiovascular disease, stroke, or various types of cancer. The benefits of a diet rich in fruits and vegetables may also result from the avoidance of ingredients of animal origin less desirable for this age, such as saturated fats and oxidized cholesterol, and may result from the intake of various antioxidant compounds, such as vitamin C and major carotenoids and dietary polyphenols [12, 25, 26].

In our study, no correlation was found between the reduction in fat consumption and the activity of antioxidant enzymes. On the other hand, it was found based on correlation and multivariate regression analysis that women who limited their consumption of fish (containing Omega 3 and 6 acids) and dairy products had lower CAT and GPx activity.

The relationship between the change in the activity of antioxidant enzymes and physical activity is not completely clear. Current research indicates that the systems of antioxidant enzymes undergo significant changes in response to acute and chronic exercise. Acute exercise may increase the activity of some antioxidant enzymes in various tissues. A small effect of physical activity on hepatic enzymes or cardiac muscle has been shown, but changes in the activity of these enzymes in skeletal muscles have been observed, especially in the case of GPx [9].

Timmerman et al. have shown that habitual physical activity in older adults may protect against reactive oxygen species damage due to higher expression of endogenous antioxidants [27].

Similar conclusions were reached by Bermudes et al., who also conducted their study on the elderly, suggesting that MVPA (moderate to vigorous physical activity) in the elderly. However, it is related to a decrease in the TAS (antioxidant enzyme activity) in women, induces an adaptive increase in antioxidant enzyme activity, and decreases lipid peroxidation in both women and men. These results suggest that at this time of life, it is not only the amount of physical activity performed that is important but also its intensity [28].

In our own study, there were no differences in the activity of antioxidant enzymes and GSH concentration in dependence from physical activity, which may be due to the fact that there was minimal physical activity in the characteristics of the study group. More than half of the women surveyed did not show any physical activity, and the part who led an active lifestyle only spent a small number of hours exercising each week.

There are more and more studies on the relationship between cigarette smoking and the activity of antioxidant enzymes in the literature. However, many of them concern pregnant women or men and women in general. In this case, it has been shown that smoking cigarettes during pregnancy and lactation results in disruption of the antioxidant balance in the woman's milk. It is also found that cigarette smoking reduces the total antioxidative capacity in colostrum and this is directly related to the number of cigarettes smoked by pregnant women [11, 26, 29]. As the literature presents, tobacco smoke contains large amounts of reactive oxygen species, which, as we know, intensify oxidative stress and cause of various pathological changes in the human body. In the serum of people who smoke, there is a significant increase in the products of oxidative damage DNA, proteins, and lipids, and at the same time a significant decrease in the activity of antioxidants. It is assumed that in one portion of inhaled tobacco smoke there are as many as 1015 ROS molecules.

These molecules include mainly semiquinone (${\text{QH}}^{ \cdot }$) radicals, but also oxygen radicals such as the hydroxyl radical, superoxide anion radical, or hydroperoxide radical, and molecules that do not belong to free oxygen radicals but are easily transformed in these forms. These include mainly hydrogen peroxide, which is a precursor of the reactive hydroxyl radical. In the human body, the concentration of hydrogen peroxide is very low, mainly due to the reductive function of CAT and GPx. Oxygen radicals react very easily with the molecules present in cigarette smoke. These molecules include, for example, hydrocarbons, and as a result of their reaction, alkoxy radicals (${\text{RO}}^{ \cdot }$) or alkyl radicals (${\text{R}}^{ \cdot }$) [30].

Dikalov et al. demonstrate that tobacco smoking-induced mitochondrial oxidative stress contributes to endothelial dysfunction and the development of hypertension. We suggest that the targeting of mitochondrial oxidative stress can be beneficial for the treatment of pathological conditions associated with tobacco smoking, such as endothelial dysfunction, hypertension, and cardiovascular diseases [31].

Based on these data, it should be assumed that in smokers, there should be a significant increase in the activity of antioxidant enzymes, which is adequate to increase the level of reactive oxygen species contained in tobacco smoke. In our study, 57% of women smoke cigarettes. However, there were no significant differences in enzyme activity and GSH concentrations in dependence from smoking cigarettes. It does not confirm, therefore, the reports of other scientists on the increase in oxidative stress as a result of smoking cigarettes. It may be associated with a well-functioning antioxidant system in the group of women surveyed, especially considering that most of them were women in the 20–35 age group.

## Conclusions

1. Increased CAT activity with age may be a sign of a large amount of hydrogen peroxide resulting from poor-functioning antioxidant systems in older age.
2. Decreased SOD activity with age may indicate inactivation of this enzyme inappropriate SOD function in the presence of excessive amounts of hydrogen peroxide, and glycation of superoxide dismutase molecules.
3. A negative correlation between superoxide dismutase activity and the BMI index may indicate a decreased enzymatic activity in obese people.
4. The concentration of GSH decreases with age, which confirms the results obtained by other researchers.

## Supplementary Information

**Additional file 1.** Supplementary tables.

## Data Availability

All data generated or analyzed during this study are included in this published article [and its additional files].
