# Supplementary material for: Activity of erythrocyte antioxidant enzymes in healthy women depends on age, BMI, physical activity, and diet
Source: J Health Popul Nutr. 2022 Aug 17;41:35. doi: 10.1186/s41043-022-00311-z (PMC9382722; doi:10.1186/s41043-022-00311-z)
Supplement: Supplementary file 1 — Additional file 1. Supplementary tables. [file 41043_2022_311_MOESM1_ESM.docx]

Table S1.Characteristics of the study group in terms of diet and smoking cigarettes

n – number of people

| **Limitation of sugar consumption** | **n** | **%** |
| --- | --- | --- |
| No | 54 | 55,1 |
| Yes | 44 | 44,9 |
| **Limitation of bread consymption** | **n** | **%** |
| No | 88 | 89,8 |
| Yes | 10 | 10,2 |
| **Limitation of fishes consumption** | **n** | **%** |
| No | 95 | 96,9 |
| Yes | 3 | 3,1 |
| **Limitation of meat consumption** | **n** | **%** |
| No | 83 | 84,7 |
| Yes | 15 | 15,3 |
| **Limitation of raw vegetables consumption** | **n** | **%** |
| No | 89 | 90,8 |
| Yes | 9 | 9,2 |
| **Limitation of raw fruits consumption** | **n** | **%** |
| No | 98 | 100,0 |
| **Limitation of dairy consumption** | **n** | **%** |
| No | 89 | 90,8 |
| Yes | 9 | 9,2 |
| **Limitation of fats consumption** | **n** | **%** |
| No | 67 | 68,4 |
| Yes | 31 | 31,6 |
| **Smoking cigarettes** | **n** | **%** |
| No | 56 | 57,1 |
| Yes | 42 | 42,9 |

Table S2. Antioxidant enzyme activity and GSH concentration (Avg - arithmetic mean, SD - standard deviation, Min - minimum value, Max - maximum value)

SOD - superoxide dismutase; CAT - catalase; GPx - glutathione peroxidase; GSH - glutathione; GST - glutathione transferase; GR - glutathione reductase

| **Antioxidant** | **Avg** | **SD** | **Min** | **Max** |
| --- | --- | --- | --- | --- |
| SOD [U/mgHb] | 0,34 | 0,20 | 0,05 | 0,9 |
| CAT [U/mgHb] | 0,31 | 0,17 | 0,01 | 0,9 |
| GPx [U/gHb] | 0,05 | 0,04 | 0,01 | 0,2 |
| GSH [µmol/gHb] | 9,87 | 2,29 | 5,34 | 19,4 |
| GST [U/gHb] | 0,04 | 0,03 | 0,00 | 0,2 |
| GR [U/gHb] | 7,37 | 11,93 | 0,04 | 77,1 |

Table S3. Antioxidant enzymes activity and GSH concentration depending on the physical activity undertaken (Avg - arithmetic mean, SD - standard deviation)

| **Parameter** | **Physical activity: No** | | **Physical activity: Yes** | | **p** |
| --- | --- | --- | --- | --- | --- |
|  | **Avg** | **SD** | **Avg** | **SD** |  |
| SOD [U/mgHb] | 0,32 | 0,18 | 0,38 | 0,22 | 0,238 |
| CAT [U/mgHb] | 0,32 | 0,15 | 0,28 | 0,19 | 0,148 |
| GPx [U/gHb] | 0,04 | 0,03 | 0,06 | 0,04 | 0,064 |
| GSH [µmol/gHb] | 9,66 | 1,97 | 10,16 | 2,66 | 0,560 |
| GST [U/gHb] | 0,05 | 0,03 | 0,04 | 0,02 | 0,075 |
| GR [U/gHb] | 5,45 | 5,63 | 9,93 | 16,81 | 0,658 |

p - statistical significance of the relationship between the physical activity undertaken, the activity of antioxidant enzymes and the concentration of GSH - non-parametric U Mann-Whitney test

Tabela S4. Antioxidant enzymes activity and GSH concentration depending on the smoking (Avg - arithmetic mean, SD - standard deviation)

| **Parameter** | **Smoking:No** | | **Smoking: Yes** | | **p** |
| --- | --- | --- | --- | --- | --- |
|  | **Avg** | **SD** | **Śr** | **SD** |  |
| SOD [U/mgHb] | 0,34 | 0,20 | 0,32 | 0,15 | 0,869 |
| CAT [U/mgHb] | 0,32 | 0,18 | 0,22 | 0,10 | 0,094 |
| GPx [U/gHb] | 0,05 | 0,04 | 0,04 | 0,03 | 0,272 |
| GSH [µmol/gHb] | 9,91 | 2,36 | 9,58 | 1,60 | 0,920 |
| GST [U/gHb] | 0,04 | 0,03 | 0,04 | 0,02 | 0,878 |
| GR [U/gHb] | 7,68 | 12,51 | 4,58 | 3,37 | 0,782 |

p - statistical significance of the relationship between the smoking undertaken, the activity of antioxidant enzymes and the concentration of GSH - non-parametric U Mann-Whitney test
